# Supplementary figures and images for: Regulation of gene transcription by thyroid hormone receptor β agonists in clinical development for the treatment of non-alcoholic steatohepatitis (NASH)
Source: PLoS One. 2020 Dec 11;15(12):e0240338. doi: 10.1371/journal.pone.0240338 (PMC7732128; doi:10.1371/journal.pone.0240338)

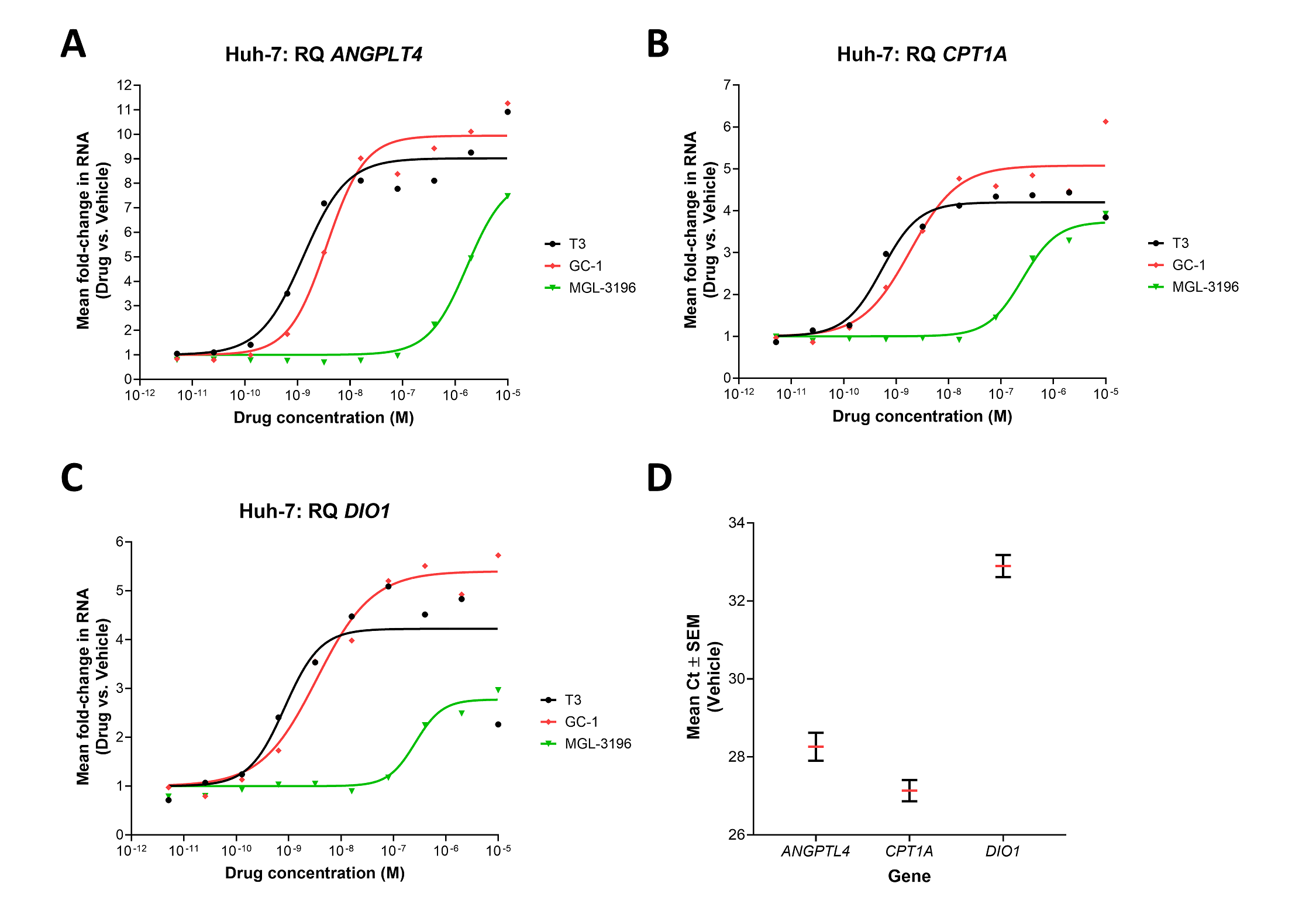

Supplement: S1 Fig — (A) ANGPLT4, (B) CPT1A, and (C) DIO1 RNA levels were quantified in the same cells described in Fig 2C and in the same manner. Results are presented as expression relative to the expression levels in control, vehicle-treated cells. Representative mean RQ values at each compound concentration and fitted dose-response curves are reported. (D) ANGPLT4, CPT1A, and DIO1 RNA levels were quantified in the same cells described in Fig 2C and in the same manner. Ct values of each gene for control, vehicle-treated groups are reported. Results are presented as mean Ct values ± SEM. (TIF) [file pone.0240338.s001.tif]

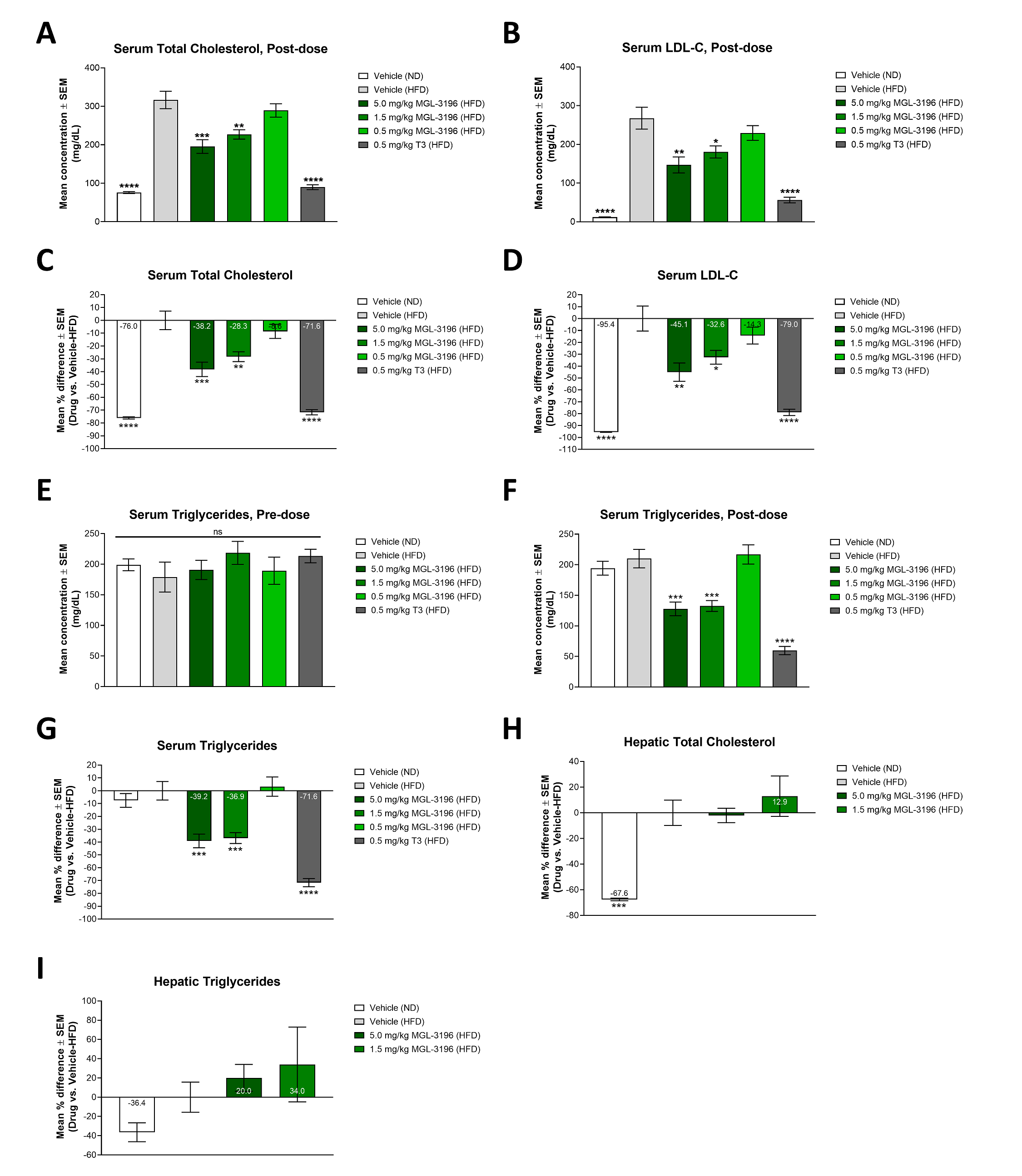

Supplement: S2 Fig — (A) Total cholesterol measurements were obtained from serum of the same rats described in Fig 3D. Total cholesterol level means ± SEM are reported. Statistical analysis was performed using Brown-Forsythe and Welch ANOVA tests and the mean of each group was compared to the mean of the HFD fed, vehicle-control group; **P < 0.01, ***P < 0.001, ****P < 0.0001. (B) LDL-C measurements were obtained from serum of the same rats described in Fig 3E. LDL-C level means ± SEM are reported. Statistical analysis was performed using Brown-Forsythe and Welch ANOVA tests and the mean of each group was compared to the mean of the HFD fed, vehicle-control group; *P<0.05, **P < 0.01, ****P<0.0001. (C) Raw total cholesterol levels reported in A) were used for calculations and are the same as those used for calculations of data reported in Fig 3D. Results are presented as percent difference from the HFD fed, vehicle-control group, post-dose. Percent change means ± SEM are reported with mean values annotated within the bars. Statistical analysis was performed using Brown-Forsythe and Welch ANOVA tests and the mean of each group was compared to the mean of the HFD fed, vehicle-control group; **P<0.01,***P<0.001, ****P < 0.0001. (D) LDL-C levels reported in B) were used for calculations and are the same as those used for calculations of data reported in Fig 3E. Results are presented as percent difference from the HFD fed, vehicle-control group, post-dose. Percent change means ± SEM are reported with mean values annotated within the bars. Statistical analysis was performed using Brown-Forsythe and Welch ANOVA tests and the mean of each group was compared to the mean of the HFD fed, vehicle-control group; *P < 0.05, **P < 0.01,****P < 0.0001. (E) Triglyceride levels were measured in the same animals described in Fig 3B. Triglyceride level means ± SEM are reported. Statistical analysis was performed using Brown-Forsythe and Welch ANOVA tests and the mean of each group was compared to the mean [file pone.0240338.s002.tif]

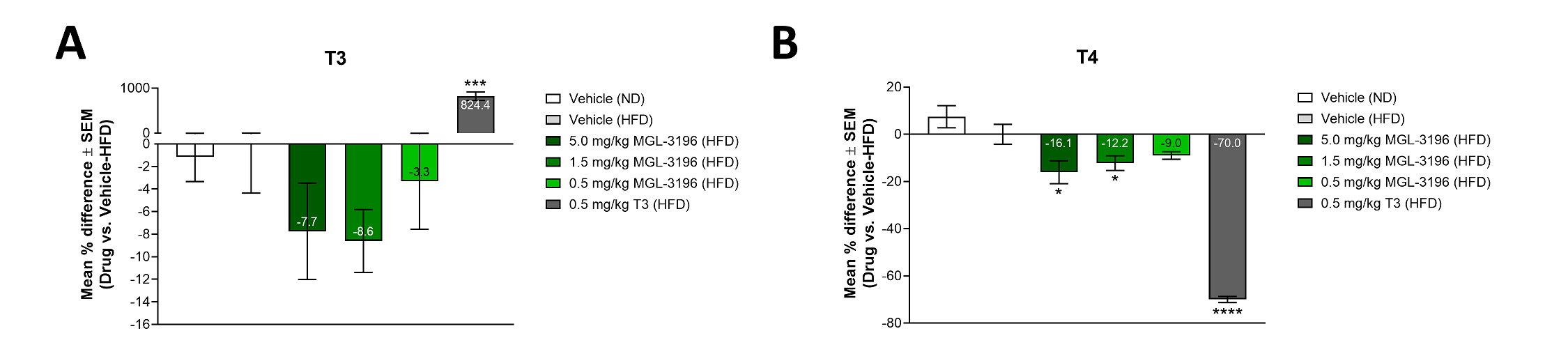

Supplement: S3 Fig — (A) T3 measurements were obtained from serum of the same rats described in Fig 3D. Results are presented as percent difference from the HFD fed, vehicle-control group, post-dose. Percent change means ± SEM are reported with mean values annotated within the bars. Statistical analysis was performed using Brown-Forsythe and Welch ANOVA tests and the mean of each group was compared to the mean of the HFD fed, vehicle-control group; ***P<0.001. (B) T4 measurements were obtained from serum of the same rats described in Fig 3D. Results are presented as percent difference from the HFD fed, vehicle-control group, post-dose. Percent change means ± SEM are reported with mean values annotated within the bars. Statistical analysis was performed using Brown-Forsythe and Welch ANOVA tests and the mean of each group was compared to the mean of the HFD fed, vehicle-control group; *P<0.05, ****P<0.0001. (TIF) [file pone.0240338.s003.tif]
